# Biomechanical analysis of different dynamic sitting techniques: an exploratory study

**DOI:** 10.1186/s12938-018-0621-2

**Published:** 2019-01-03

**Authors:** Chun-Ting Li, Yen-Nien Chen, Yen-Ting Tseng, Kuen-Horng Tsai

**Affiliations:** 1 Graduate Institute of Mechatronic System Engineering, National University of Tainan, No. 33, Sec. 2, Shu-Lin St., West Central Dist., Tainan, 70005 Taiwan; 2 Department of BioMedical Engineering, National Cheng Kung University, No.1, University Rd., East Dist., Tainan, 70101 Taiwan; 3 Department of Orthopedics, Show-Chwan Memorial Hospital, No. 542, Sec. 1, Chung-Shan Rd., Changhua, 50008 Taiwan

**Keywords:** Pressure ulcers, Wheelchair, Dynamic sitting, Interface pressure

## Abstract

Prolonged static sitting in wheelchairs increases the risk of pressure ulcers. This exploratory study proposed three dynamic sitting techniques in order to reduce the risk of developing pressure ulcer during wheelchair sitting, namely lumbar prominent dynamic sitting, femur upward dynamic sitting, and lumbar prominent with femur upward dynamic sitting. The purpose of this study was to analyze the biomechanical effects of these three techniques on interface pressure. 15 able-bodied people were recruited as subjects to compare the aforementioned sitting techniques in a random order. All parameters, including dynamic contact area, dynamic average pressure, and dynamic peak pressure on backrest and seat were measured and compared. In result, when compared with lumbar prominent dynamic sitting, femur upward dynamic sitting and lumbar prominent with femur upward dynamic sitting appeared to yield significantly lower dynamic average and peak pressure on the back part of seat, and significantly higher dynamic average and peak pressure on the front part of seat. This study can serve as a reference point for clinical physicians or wheelchair users to identify a suitable dynamic sitting technique.

## Introduction

Wheelchairs are generally used by patients suffering from lower limb disorders. Studies have indicated that a prolonged static sitting in wheelchairs increases the risk of pressure ulcers [1–3]. It is most commonly induced by long-term stress concentration on ischial tuberosities, coccyx, or bony prominences can cause blockages in blood vessels of the surrounding tissues, affect the supply of nutrition to cells, and lead to cell necrosis [1, 2, 4, 5]. The wheelchair seating system is a crucial factor affecting the buttocks pressure [6–12].

Previous studies have reported that lumbar supports and cushions correct the problem of stress concentration at the ITs by redistributing stress on the back, buttocks, and thighs [6, 7]. However, these assistive devices cannot reduce the stress load caused by prolonged static sitting. Some studies have already proposed dynamic sitting techniques (DSTs) that are creatively designed with special purposes, experiments have also proven that these seating systems are able to periodically change sitting postures and reduce prolonged static sitting loads [13–15]. Nonetheless, the optimal movement strategy of DST to reduce the risk of developing pressure ulcers is still being debated.

**Fig. 1:**
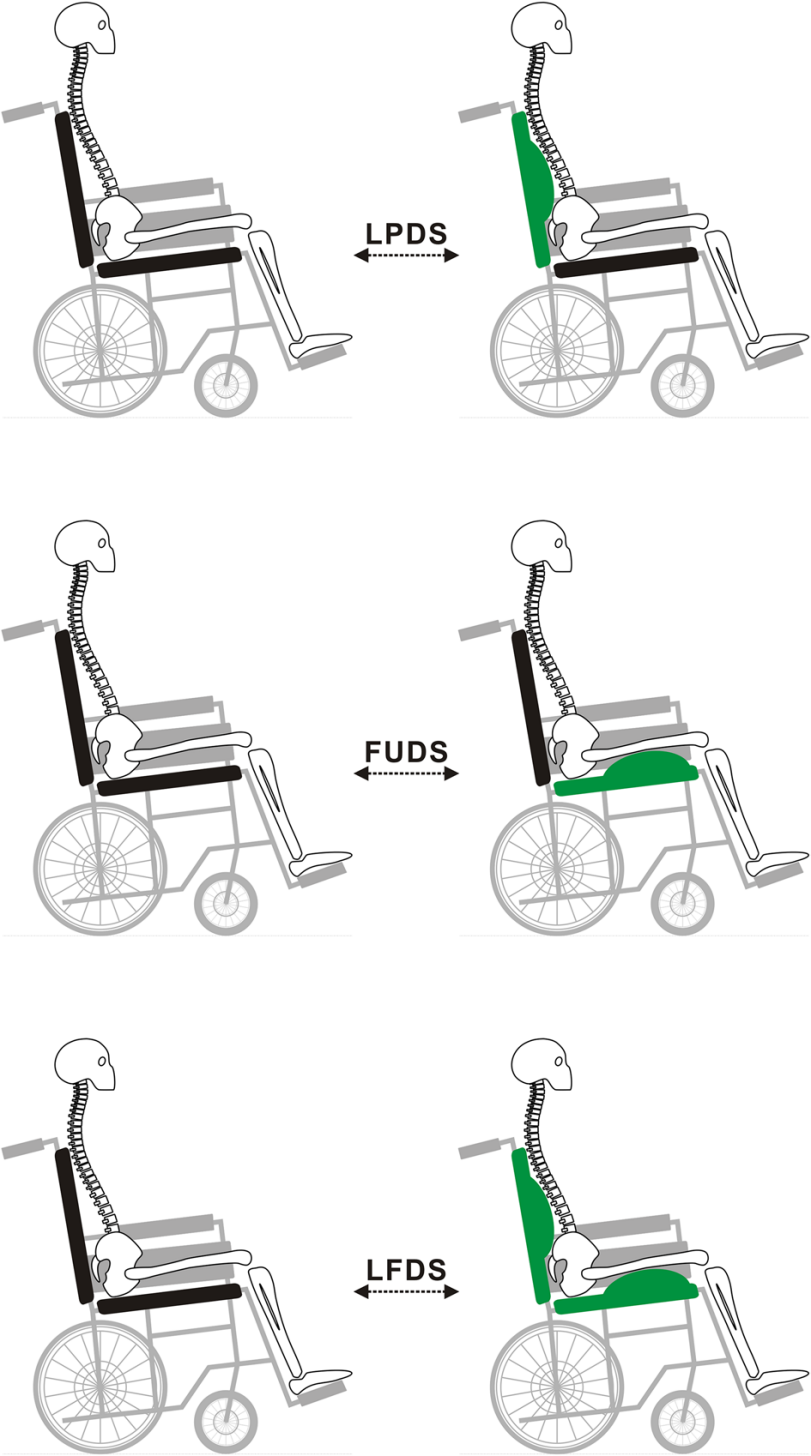
Three different dynamic sitting techniques. Including the lumbar prominent dynamic sitting (LPDS), femur upward dynamic sitting (FUDS), and lumbar prominent with femur upward dynamic sitting (LFDS)

Although lumbar supports and cushions are the most common static form of pressure-relieving assistive devices for wheelchair users, studies on their dynamic design and interface pressure measurement are lacking. Therefore, this exploratory study engaged in the dynamic design of above-mentioned assistive devices, and developed three innovative DSTs, namely lumbar prominent dynamic sitting (LPDS), femur upward dynamic sitting (FUDS), and lumbar prominent with femur upward dynamic sitting (LFDS), as illustrated in Fig. 1. The purpose of this study was to analyze the biomechanical effects of LPDS, FUDS, and LFDS on interface pressure.

## Methods

### Subjects

Fifteen able-bodied people were recruited to participate in this exploratory study (8 men, 7 women; age, 22.5 ± 1.8 years; weight, 65.2 ± 10.6 kg; height, 168.5 ± 8.9 cm; body mass index, 22.8 ± 2.6 kg/m^2^). Subjects with preexisting musculoskeletal disorders and spinal pathologies were excluded. All subjects read and signed an informed consent form that explained the study objective and experimental protocol. This study was approved by the Institutional Review Board of National Cheng Kung University Hospital.

### Experimental wheelchair

The researcher of this study designed an experimental wheelchair, which was installed with airbags providing adjustable support for lumbar and femur areas. The size of each airbag was 40 × 23 cm^2^. When filled with air, the airbags were 4 cm thick. A customized microprocessor was used to adjust the extent and cycle period of filling or deflating each airbag to periodically change the sitting postures. A 1-cm-thick foam pad was installed to the backrest and seat to minimize the discomfort caused by the surface discontinuity between the skin contact with the backrest and seat.

### Experimental protocol

Before the experiment started, each subject was first asked to rest their upper body on the backrest and relax their arms on both sides. Moreover, they had to keep their thighs parallel to the ground, feet approximately shoulder-width apart and firm on the footrest, and eyes looking straight ahead [16, 17]. Afterward, the sequence of three DSTs was randomly drawn by each subject (illustrated in Fig. 1): (1) LPDS: An airbag providing adjustable support for lumbar area was placed at L3 (on the subject). The airbag configuration was periodically switched between deflated (0-cm-thick) and filled (4-cm-thick), alternating every 5 min, the total period of experiment lasted for 20 min. (2) FUDS: An airbag providing adjustable support for femur area was placed at the middle of the subject’s thighs. The airbag configuration was periodically switched between deflated (0-cm-thick) and filled (4-cm-thick), alternating every 5 min, the total period of experiment lasted for 20 min. (3) LFDS: This technique is a combination of the previous 2 techniques simultaneously. Between each DST experiment, the subject took a 5-min break during which they could stand up and walk around.

### Data recordings and analysis

Two pressure mapping mats (Body Pressure Measurement System; Tekscan Inc, South Boston, Massachusetts, USA) were placed over the surface of the backrest and seat to measure the pressure distribution along the human-wheelchair interface. The pressure mapping mats comprised 2016 (48 × 42) measuring cells. Each measuring cell had a dimension of 10.16 × 10.16 mm^2^. The interface pressure parameters were calculated using the body pressure measurement system research software (BPMS, version 7.02C; Tekscan Inc, South Boston, Massachusetts, USA). Data were sampled at the frequency of 30 Hz. From the interface pressure recordings, the dynamic contact area (DCA), dynamic average pressure (DAP), and dynamic peak pressure (DPP) on the whole backrest (B-DCA, B-DAP, and B-DPP), the entire seat (S-DCA, S-DAP, and S-DPP), the back part of seat (BS-DCA, BS-DAP, and BS-DPP), and the front part of seat (FS-DCA, FS-DAP, and FS-DPP) were calculated. Each interface pressure parameter is given as the averaging value over the entire 20 min sitting trial.

### Statistical analysis

The Statistics Package for the Social Sciences (SPSS, version 17; SPSS Institute, Chicago, IL, USA) was used for all statistical analyses. Firstly, the whole backrest (B-DCA, B-DAP, and B-DPP) and the entire seat (S-DCA, S-DAP, and S-DPP) were compared between the different DSTs (LPDS, FUDS and LFDS) by using a Friedman test. A post hoc test, Wilcoxon signed-rank test was used for detecting significant differences in the dependent variables across the tests. Secondly, the back part of seat (BS-DCA, BS-DAP, and BS-DPP) and the front part of seat (FS-DCA, FS-DAP, and FS-DPP) were compared between the three DSTs by using the same statistical methods as above. The statistical significance was set at *P* < 0.05.

## Results

The interface pressure measurements on both the whole backrest and the entire seat are shown in Table 1. When compared with LPDS, FUDS appeared to yield significantly lower B-DAP (*P* = 0.036) values, but no significant differences in B-DCA, B-DPP, S-DCA, S-DAP, and S-DPP values were observed. When compared with LPDS, LFDS appeared to yield significantly higher B-DAP (*P* = 0.033) and lower S-DPP (*P* = 0.011) values, but no significant differences in B-DCA, B-DPP, S-DCA, and S-DAP values were observed. When compared with FUDS, LFDS appeared to yield significantly higher B-DCA (*P* = 0.005), B-DAP (*P* = 0.010), and B-DPP (*P* = 0.047) values, but no significant differences in S-DCA, S-DAP, and S-DPP values were observed.

**Table 1:** Results of interface pressure measurement on both the whole backrest and the entire seat

DSTs	B-DCA (cm^2^)	B-DAP (kPa)	B-DPP (kPa)	S-DCA (cm^2^)	S-DAP (kPa)	S-DPP (kPa)
LPDS	143.80 ± 61.70	2.93 ± 0.61	5.19 ± 1.63	837.80 ± 193.03	6.28 ± 0.85	41.77 ± 12.19
*P* ^*^	0.053	*0.036*	0.244	0.842	0.975	0.056
*P* ^*†*^	0.222	*0.033*	0.084	0.363	0.470	*0.011*
FUDS	109.01 ± 67.56	2.55 ± 0.37	4.71 ± 2.59	823.06 ± 140.88	6.24 ± 0.61	37.58 ± 10.55
*P* ^*‡*^	*0.005*	*0.010*	*0.047*	0.629	0.648	0.807
LFDS	176.02 ± 78.54	3.18 ± 0.71	6.04 ± 1.97	815.06 ± 184.13	6.38 ± 0.81	37.65 ± 12.83
*P* ^*§*^	*0.022*	*0.006*	*0.028*	0.936	0.948	*0.006*

Comparison of mean dynamic contact area (DCA), dynamic average pressure (DAP), and dynamic peak pressure (DPP) on both the whole backrest (B-DCA, B-DAP, and B-DPP) and the entire seat (S-DCA, S-DAP, and S-DPP) across three dynamic sitting techniques (DSTs), which include lumbar prominent dynamic sitting (LPDS), femur upward dynamic sitting (FUDS), and lumbar prominent with femur upward dynamic sitting (LFDS). Each interface pressure parameter is given as the averaging value over the entire 20 min sitting trial. Values are mean ± standard deviation (N = 15)

^*^P (*W*) is the significance difference as compared LPDS with FUDS

^*†*^*P* (*W*) is the significance difference as compared LPDS with LFDS

^*‡*^*P* (*W*) is the significance difference as compared FUDS with LFDS

^*§*^*P* (*F*) is the significance difference as compared between the all DSTs

**Fig. 2:**
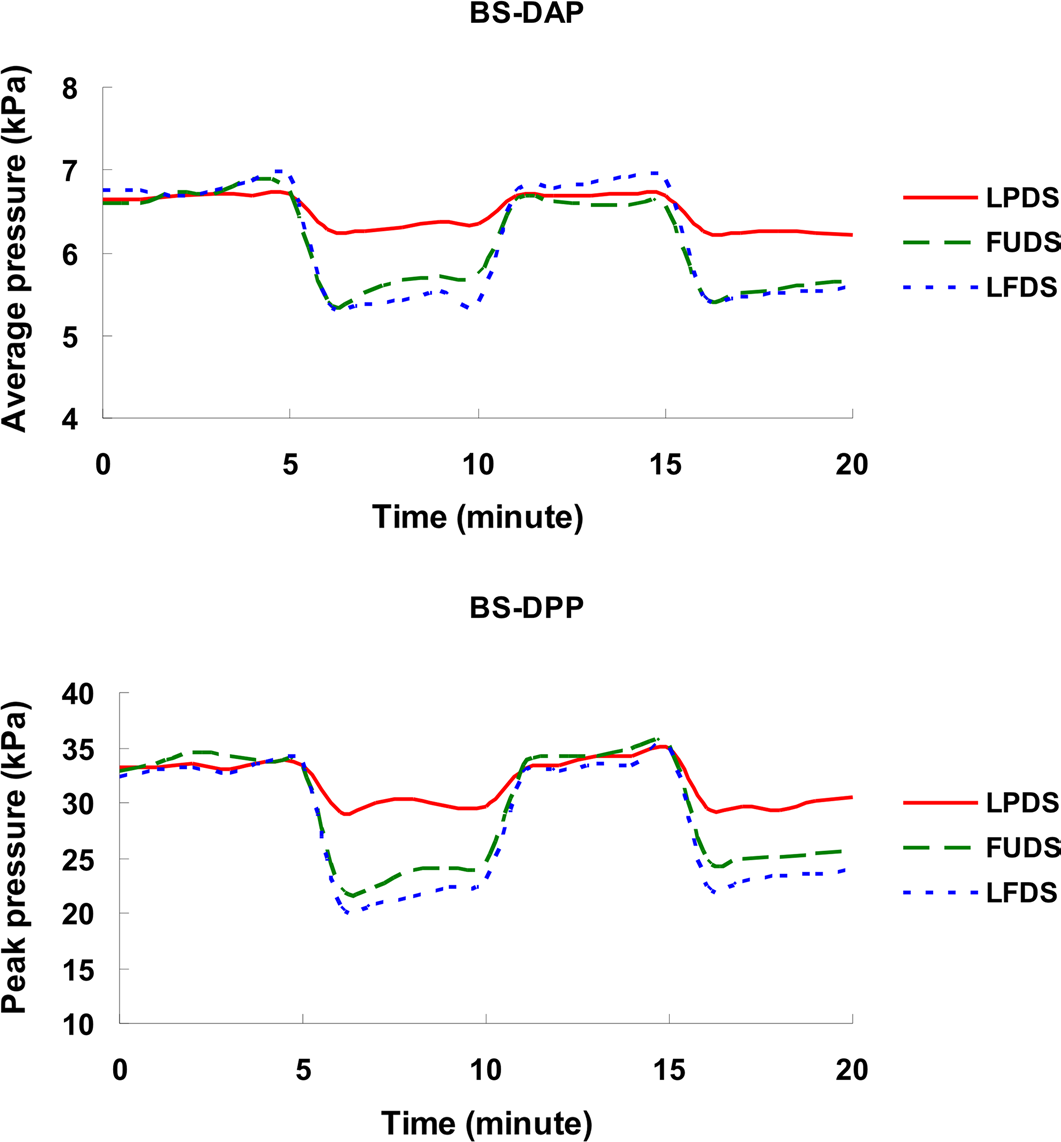
Representative dynamic average pressure (DAP) and dynamic peak pressure (DPP) on the back part of seat (BS-DAP and BS-DPP) for a participant during lumbar prominent dynamic sitting (LPDS), femur upward dynamic sitting (FUDS), and lumbar prominent with femur upward dynamic sitting (LFDS) trial conditions

The interface pressure measurements on both the back part of seat and the front part of seat are shown in Table 2. Representative BS-DAP and BS-DPP for a participant during LPDS, FUDS, and LFDS trial conditions are shown in Fig. 2. When compared with LPDS, FUDS appeared to yield significantly lower BS-DAP (*P* = 0.005) and BS-DPP (*P* = 0.009) values, and higher FS-DAP (*P* = 0.001) and FS-DPP (*P* = 0.001) values, but no significant differences in BS-DCA and FS-DCA values were observed. When compared with LPDS, LFDS appeared to yield significantly lower BS-DCA (*P* = 0.015), BS-DAP (*P* = 0.002), and BS-DPP (*P* = 0.009) values, and higher FS-DAP (*P* = 0.001) and, FS-DPP (*P* = 0.001) values, but no significant differences in FS-DCA values were observed. When FUDS was compared with LFDS, no significant differences in all interface pressure parameters of the back and front part of seat were observed.

**Table 2:** Results of interface pressure measurement on both the back part of seat and the front part of seat

DSTs	BS-DCA (cm^2^)	BS-DAP (kPa)	BS-DPP (kPa)	FS-DCA (cm^2^)	FS-DAP (kPa)	FS-DPP (kPa)
LPDS	527.13 ± 64.00	8.11 ± 0.99	41.28 ± 12.35	342.13 ± 95.11	3.47 ± 0.56	6.55 ± 1.52
*P* ^*^	0.173	*0.005*	*0.009*	0.496	*0.001*	*0.001*
*P* ^*†*^	*0.015*	*0.002*	*0.009*	0.712	*0.001*	*0.001*
FUDS	517.60 ± 80.16	7.13 ± 0.86	35.77 ± 10.80	350.00 ± 61.37	4.73 ± 0.42	9.97 ± 1.19
*P* ^*‡*^	0.069	0.460	0.875	0.932	0.154	0.065
LFDS	495.73 ± 59.34	7.37 ± 1.14	36.88 ± 13.22	347.07 ± 83.07	4.88 ± 0.61	10.73 ± 1.66
*P* ^*§*^	*0.031*	*0.001*	*0.004*	0.819	*< 0.001*	*< 0.001*

Comparison of mean dynamic contact area (DCA), dynamic average pressure (DAP), and dynamic peak pressure (DPP) on both the back part of seat (BS-DCA, BS-DAP, and BS-DPP) and the front part of seat (FS-DCA, FS-DAP, and FS-DPP) across three dynamic sitting techniques (DSTs), which include lumbar prominent dynamic sitting (LPDS), femur upward dynamic sitting (FUDS), and lumbar prominent with femur upward dynamic sitting (LFDS). Each interface pressure parameter is given as the averaging value over the entire 20 min sitting trial. Values are mean ± standard deviation (N = 15)

^*^P (*W*) is the significance difference as compared LPDS with FUDS

^*†*^*P* (*W*) is the significance difference as compared LPDS with LFDS

^*‡*^*P* (*W*) is the significance difference as compared FUDS with LFDS

^*§*^*P* (*F*) is the significance difference as compared between the all DSTs

## Discussion

This study examined and quantified the biomechanical effects of three DSTs, namely LPDS, FUDS, and LFDS, by measuring the interface pressure. The results of the experiments revealed that FUDS and LFDS periodically changed the interface pressure on the buttocks that can be used to reduce the risk of developing pressure ulcers. LPDS yielded the significantly lowest performance in interface pressure on the buttocks.

The weight of a wheelchair user is primarily supported by the backrest and seat. In addition, because the buttocks support most of the body weight, the stress of body weight is primarily focused on the ischial tuberosities and the surrounding soft tissues, increasing the likelihood of developing pressure ulcers there [8, 9, 18, 19]. Previous studies have indicated that increasing the stress load between the backrest and the front part of seat can assist in reducing the stress on the ischial tuberosities [8, 9]. The results of the interface pressure measurements illustrated that the three DSTs yielded significant differences in B-DAP values. LFDS yielded the significantly highest B-DAP values, LPDS the significantly medium, and FUDS the significantly lowest. Consequently, LFDS is more effective than LPDS and FUDS at shifting the sitting load from the seat to the backrest when dynamic alteration process. Moreover, compared with LPDS, FUDS and LFDS yielded significantly lower BS-DAP and BS-DPP values, and significantly higher FS-DAP and FS-DPP values. Regarding these parameters, there were no significant differences between FUDS and LFDS. Consequently, FUDS and LFDS are more effective than LPDS at shifting the sitting load from the back part of seat to the front part of seat when dynamic alteration process; periodically reduce the interface pressure on the buttocks.

In study limitations, the subjects in this study were able-bodied people rather than people with lower-limb disorders, because we concerned about the imposed physiological loads and danger on people with lower-limb disorders for this experiment that required extended processes and multiple sessions of repositioning. In addition, this study focused on reducing the risks of pressure ulcer from a preventive concept and expected participants who had healthy and functional torsos to reach the experiment target. Therefore, we decided to recruit able-bodied people who were not wheelchair users and eliminated those diagnosed with musculoskeletal disorders and spinal pathologies. If applying the study results to wheelchair users, their different pathological characteristics should be considered to ensure feasibility.

In conclusion, the results of this study suggested that FUDS and LFDS can be used to periodically change the distribution of interface pressure on the buttocks. This study can serve as a reference point for clinical physicians or wheelchair users to identify a suitable DST. Thus, further studies should focus on identifying the most adequate adjustment degree and cycle period of the dynamic alteration process.
